# MT1-MMP-dependent ECM processing regulates laminB1 stability and mediates replication fork restart

**DOI:** 10.1371/journal.pone.0253062

**Published:** 2021-07-08

**Authors:** Varsha Thakur, Juliano Tiburcio de Freitas, Yuan Li, Keman Zhang, Alyssa Savadelis, Barbara Bedogni

**Affiliations:** 1 Department of Dermatology, University of Miami Miller School of Medicine, Miami, FL, United States of America; 2 Department of Biochemistry, Case Western Reserve University, Cleveland, OH, United States of America; Centre National de la Recherche Scientifique, FRANCE

## Abstract

Radiotherapy remains a mainstay of treatment for a majority of cancer patients. We have previously shown that the membrane bound matrix metalloproteinase MT1-MMP confers radio- and chemotherapy resistance to breast cancer via processing of the ECM and activation of integrinβ1/FAK signaling. Here, we further discovered that the nuclear envelope protein laminB1 is a potential target of integrinβ1/FAK. FAK interacts with laminB1 contributing to its stability. Stable laminB1 is found at replication forks (RFs) where it is likely to allow the proper positioning of RF protection factors, thus preventing RF degradation. Indeed, restoration of laminB1 expression rescues replication fork stalling and collapse that occurs upon MT1-MMP inhibition, and reduces DNA damage in breast cancer cells. Together, these data highlight a novel mechanism of laminB1 stability and replication fork restart via MT1-MMP dependent extracelluar matrix remodeling.

## Introduction

Over 470,000 cancer patients receive radiotherapy each year in the United States [1], equal to as many as half of all patients under care [2]. Radiotherapy is used to treat the cancer, but also to reduce cancer side effects, which may substantially affect a patient’s quality of life. In breast cancer, the second leading cause of cancer death in women [3], radiation therapy is a critical component in the management of invasive disease [4], while in triple-negative breast cancers (TNBCs), which do not have available targeted therapy options as a standard of care, combined radiation and chemotherapy is the main therapeutic option [5].

Resistance to radiotherapy however, represents a major hurdle to therapy response in cancer patients, including breast cancer patients. Radiation induced DNA damage and cytotoxicity is a main anti-cancer mechanism, but at the same time, cells undergoing radiation activate several pro-survival signaling pathways (e.g. AKT, ERK, ATM/ATR) to counteract the damage, thus leading to the suppression of apoptosis; cell cycle arrest; and the activation of DNA repair mechanisms which together contribute to radioresistance [6,7].

We have previously identified a novel mechanism of radioresistance involving extracellular matrix remodeling by the metalloproteinase MT1-MMP [8]. MT1-MMP is a zinc-dependent Matrix Metalloproteinase bound to the plasma-membrane involved in basement membrane and extracellular matrix (ECM) remodeling during cancer cell invasion [9,10]. MT1-MMP also promotes tumorigenesis by activating effector MMPs (e.g. MMP2, MMP13) and growth factors (e.g. EGF, CD44, Notch1) [11–13]. Surprisingly, however, we have demonstrated that, aside from these canonical functions in cancer cell invasion and metastases, MT1-MMP confers radio- and chemotherapy resistance to breast cancer [8]. MT1-MMP promotes radio- and chemoresistance through cleavage of the ECM. ECM processing then leads to integrinβ1/FAK activation that promotes replication fork (RF) stability [8].

Here, we further show that MT1-MMP confers radio/chemo-resistance by regulating the stability of laminB1. Lamins are evolutionarily conserved members of a large family of intermediate filaments (IF) [14]. Lamins are major structural components of the nucleus localized predominantly at the nuclear rim, with laminB1 tethered to the membrane via a farnesyl group. Studies in cells from progeria patients in which type A lamins are mutated, show that these cells have defective DNA repair mechanisms and tend to accumulate DNA damage leading to accumulation of chromosomal instability [14,15]. Moreover, laminB1 inhibition has been shown to stall and collapse replication forks leading to DSBs [16]. Here we show that reduced MT1-MMP results in reduced laminB1 at replication forks and consequent reduction in RF protection factors associated with RFs (e.g. RAD51). Conversely, restoration of laminB1 levels reduces DNA damage and replication fork stalling triggered by MT1-MMP inhibition. Together, we uncovered a novel mechanism of DNA Damage Repair linking the extracellular matrix to replication fork stability via modulation of the nuclear intermediate filament envelope protein laminB1.

## Materials and methods

### Cell lines, plasmids and reagents

MDA-MB-231 cells were obtained form ATCC and grown in RPMI-1640 supplemented with 10% FBS and maintained at 37°C with 5% CO2. MDA-MB-231 cells were authenticated in 2013 by STR profiling (BDC Molecular Biology Core Facility, University of Colorado). Cells were tested for mycoplasma every month (MycoAlert^TM^ Mycoplasma Detection Kit, Lonza). Doxorubicin hydrochloride (Kayman Chemicals) was dissolved in DMSO (Sigma Aldrich). mRNA silencing of MT1-MMP was performed using shRNA TRCN0000050855 (Sigma Aldrich) against MT1-MMP, which was previously characterized and validated [13,17,18]. shRNAs TRCN00000297156 and TRCN0000029272 (Sigma Aldrich) were used to inhibit laminB1 expression. Wild Type LaminB1 was kindly provided by Dr. Shelly L. Berger, University of Pennsylvania, and introduced into pLM-CMV-Ha-puro-PL3 lentiviral plasmid as previously described [8,13,17]. The Focal Adhesion Kinase inhibitor (VS4718) [19–21] was purchased from Selleck Chemicals, and was used at 400 nM in DMSO.

### Real-Time PCR

cDNA was synthesized from total RNA using the SuperScript first-strand synthesis system for reverse transcription-PCR (RT-PCR) (Invitrogen). cDNA was used for PCR amplification with SYBR green PCR master mix (Roche). The following primers were used: human β-actin: forward (5’-TGCGTGACATTAAGG AGAA-3’), reverse (5’-AAGGAAGGCTGGAAG AGT-3’); human MT1-MMP forward (5’-CTCCCTCGGCTCGGCCCAAA-3’), reverse (5’-CGCCTCATGGCCTTCATGGTGTCT-3’); human laminB1 forward (5’-CCAGGGAAGAACTGATGGAA-3’), reverse (5’-CAGCTGTTGCTGCATTTGAT-3’).

### Western blotting

Cell seeding (2x10^6^ cells per 10 cm dish), collection of protein and Western blot methods were as previously described [22] Membranes were probed with the following antibodies: anti-MT1-MMP (LEM-2/15.8, EMD Millipore, MA); anti γH2AX (clone JBC301, EMD Millipore, MA); anti laminA/C (clone EPR4100, Abcam); anti laminB1 (clone B10, Santa Cruz Biotechnology); anti RAD51 (clone EPR4030(3), Abcam); anti total and phospho-FAK^Y397^ (total: clone EP695Y; phosphorylated: clone EP2160Y, Abcam); anti-p-ChK1 (clone 133D3), anti p-ChK2 (clone C13C1), anti p-RPA32 (clone E5A2F) were from Cell Signaling Technology; β-actin (clone C4) and GAPDH (clone 0411) were from Santa Cruz Biotechnology.

### qGEL 3D matrix assay (degradable and non-degradable matrixes)

Cell suspension of 10^6^ cells per 100 μL was combined with 400 μL of HEPES with qGEL Lyophilized Powder (formulation IDs: NSC4QA432R and NSC4EN562R [8,23], qGel Bio). The mixture was incubated at 37°C, 5% CO_2_ for 30 minutes until a solid matrix was formed with cells embedded inside. Media (2 mL) was then added. Cell lysates were obtained as described above.

### Coimmunoprecipitation

2x10^6^ MDA-MB-231 cells were grown on 10-cm plates for 48 hours. Cells were then washed with ice cold PBS and resuspended in RIPA buffer (50mM Tris HCl pH 8, 150 mM NaCl, 1% NP-40, 0.5% sodium deoxycholate) along with protease inhibitors. Lysed cells were centrifuged at 4°C at 14,000 rpm for 10 min and the supernatant was immunoprecipitated overnight at 4°C with either a laminB1 (Santa Cruz Biotechnology) or FAK (Abcam) antibodies. IgG was used as negative IP control. The immunoprecipitated fractions were analyzed by immunoblotting using anti-laminB1 and anti FAK antibodies.

### Colony formation assay

Irradiation of cells was performed with a ^137^Cs irradiator (Shepherd). A total of 500 to 10,000 cells per plates were stained after 10 days with 0.1% crystal violet. Assays were done ≥3 times with individual samples in triplicate. To determine the extent of radiosensitization, the DEF (Dose Enhancement Factor) was determined by the ratio between the radiation or DRX dose alone at 0.1 survival fraction versus radiation or DRX + shRNA against laminB1 or MT1-MMP +/- laminB1 expression at the same survival fraction (DEF>1 = sensitizer; DEF<1 = radioprotector). Data presented are the mean of three experimental repeats.

### Immunofluorescence

Cells were fixed in 3% formaldehyde, air dried and washed twice in PBS, then blocked using goat serum. Sections were then incubated O/N at 4C with the primary antibodies. Sections were washed three times in PBS prior to incubation with secondary antibodies for 30 minutes at RT. An anti γH2AX (S139) antibody (JBC301, EMD Millipore, MA) with secondary Alexa-Fluor 594 anti-mouse (A11032; Invitrogen) was used to stain foci. Immunofluorescence was observed at X100 or X60 magnification using NIKON 90i fluorescence microscope (photometric cooled mono CCD camera; Nikon), and foci were counted from at least 50 cells. Data are the mean of three experimental repeats. Anti laminB1 (clone B10, Santa Cruz Biotechnology); anti RAD51 (clone EPR4030(3), Abcam); anti MT1-MMP (clone EP1264Y, Abcam); anti-BrdU (B44, BD Biosciences), anti FAK (clone 2C5B9, Proteintech) were detected with FITC or Alexa-Fluor 594 conjugated secondary antibodies. All slides were mounted with Vectashield containing DAPI (Vector Laboratories).

### Comet assay

Comet assays were performed as directed (Trevingen). Briefly, 1×10^6^ cells were irradiated or left untreated, then cultured for up to 24 hrs. Cell suspensions of equal number of cells were mixed with pre-melted low melting agarose, mixed and plated on glass slides provided in the kit and left at 4°C for 30 minutes to allow solidification of the agarose and then immersed in cold lysis buffer for 2 hours to ensure complete lysis. Electrophoresis was carried out at 21 volts for 45 minutes using neutral electrophoresis buffer (1×TBE). Slides were fixed with 70% ethanol and then dried at 37°C overnight. DNA was stained using 1:10,000× Syber green DNA stain. Comets were imaged at 10X objective. Comet analysis was done using Comet Score (TriTek). A minimum 50 comets were included per condition and done in duplicate. At least two repeats were done for each experiment. Data are the mean of all experimental repeats.

### DNA fiber assay

to determine replication fork restart, cells were pulse-labeled with 50 μmol/L IdU (Sigma-Aldrich, followed by treatment with hydroxyurea to stall replication, then pulsed-labeled 200 μmol/L CldU (Sigma–Aldrich). To assess replication fork stability, cells were pulse with IdU and CldU following HU at the doses and times indicated. Cell suspensions were then mixed with lysis buffer [0.5% SDS, 200 mmol/L Tris-HCl (pH 7.4), 50 mmol/L EDTA] and dropped and spread onto an uncoated glass slide and let dry. DNA spreads were fixed with a 3:1 solution of methanol-acetic followed by 70% ethanol at 4°C for 1 hour. DNA was denatured with 2.5 mol/L HCl for 30 minutes at 37°C. Slides were blocked in 1% BSA and then incubated with mouse anti-BrdU antibody (BD Biosciences) and rat anti-CldU antibody (Abcam). Alexa Fluor 594, or Alexa Fluor 488 labeled secondary antibodies were used (Thermo Fisher Scientific). Replication fibers were viewed at 100X magnification on a NIKON 90i fluorescence microscope (photometric cooled mono CCD camera; Nikon). Red and green track signals as well as length were measured using ImageJ software (NCI/NIH), as previously described [24]. Experiments were performed at least twice.

### Statistical analysis

Statistical significance was determined using the Student’s *t* test with a significant difference being at least p<0.05. Error bars represent the standard deviations of the mean. *P* values are either directly shown in the figure or represented by one or more star symbols. The *p* value of each star symbol is reported in the figure legend. All experiments were repeated at least three times, unless otherwise stated in the figure legends.

## Results and discussion

### MT1-MMP modulates the levels of laminB1 likely via processing of the ECM and activation of integrinβ1/FAK

We have previously shown that MT1-MMP inhibition triggers DNA replication fork stalling and collapse resulting in formation of double strand breaks (DSBs), and that the endogenous increase in DNA damage renders cells more susceptible to further genotoxic stresses [8]. Intriguingly, the phenotype associated with MT1-MMP inhibition is reminiscent of cells depleted of laminB1 [16,25–28], whereby laminB1 inhibition has been shown to cause stalling and collapse of replication forks leading to DSBs [16]. We therefore hypothesized that the replication stress and accumulation of DSBs caused by MT1-MMP inhibition might be through reduction of lamins.

**Fig 1:**
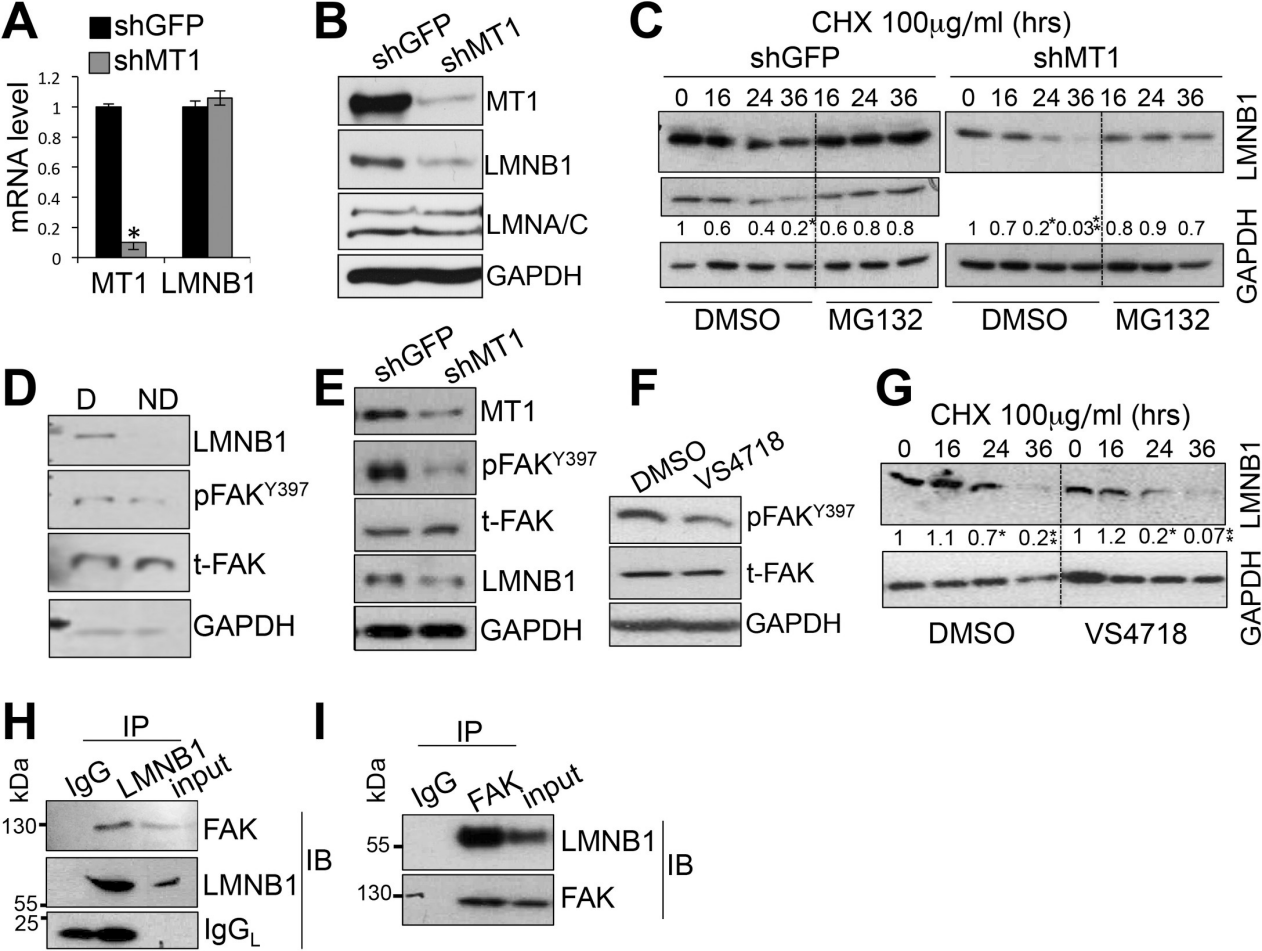
MT1-MMP affects laminB1 stability via integrinβ1/FAK. mRNA levels (**A**), measured by quantitative RT-PCR are the ratio between laminB1 or MT1-MMP mRNA and b-actin mRNA. Protein levels (**B**), assessed by western blotting, of laminB1 (LMNB1), lamina/C (LMNA/C) and MT1-MMP (MT1) in MDA-MB-231 cells expressing shGFP or shMT1-MMP. **C)** Western blotting showing levels of laminB1 in MDA-MB-231 cells expressing either shGFP or shMT1-MMP treated with 100 μg/ml cycloheximide for the time indicated and with 50 μg MG132 (proteasome inhibitor). Rates of degradation are shown. These are the mean of two independent experiments. Values are the ratio between band intensity of LMNB1 and GAPDH, evaluated by ImageJ. Time 0 (T0) was normalized to 1. *p<0.05,**p<0.001, Student’s *t* test **D)** Levels of LMNB1, phspho-FAK^Y397^, total FAK and GAPDH, used as loading control, assessed by western blotting in total lysates of cells seeded in degradable (D) or non-degradable (ND) hydrogels. **E)** LMNB1, FAK^Y397^ and total FAK assessed by western blotting in total lysates of cells expressing shMT1-MMP. **F)** Levels of phospho-FAK^Y397^, assessed by western blotting, in MDA-MB-231 cells treated for 36 hours with the FAK inhibitor VS4718 used at 400nM. **G)** Inhibition of FAK activity by VS4718 accelerates LMNB1 degradation. LaminB1 levels were assessed by western blotting in cells treated with DMSO or VS4718 in the presence of cycloheximide (100μg/ml), for the time indicated. Rates of degradation are shown and are the mean of two independent experiments. Values are the ratio between band intensities of LMNB1 and GAPDH, measured by ImageJ. Time 0 (T0) was normalized to 1. *^,^**p<0.001, Student’s *t* test. **H-I)** Representative co-immunoprecipitation assays–(H) IP: Immuno-precipitation of laminB1. IB: Immunoblotting with anti FAK and anti LMNB1. LaminB1 enrichment versus input: 4.6 +/- 1; FAK enrichment versus input: 6.4 +/- 1.05. (I) IP: Immuno-precipitation of FAK. IB: Immunoblotting with anti FAK and anti LMNB1. LaminB1 enrichment versus input: 5.5 +/- 0.4; FAK enrichment versus input: 2.9 +/- 0.2. IgG was used as negative control. IgG_L_ in Fig 1H is the light chain of IgG. IPs were performed twice.

While MT1-MMP depletion did not affect laminA and C levels, it did reduce laminB1 protein levels, without changing laminB1 mRNA (Fig 1A and 1B). Of note, cell senescence has been shown to cause reduced LaminB1 levels in fibroblasts [29]. However, we have previously demonstrated that MT1-MMP inhibition does not cause senescence in TNBC cells [8]. Thus, we posited that MT1-MMP might regulate laminB1 protein stability, since no change in laminB1 mRNA levels were observed in MT1-MMP depleted cells. Indeed, we found that the rate of laminB1 degradation was greater in shMT1-MMP expressing cells than in controls (Fig 1C). However, the levels of laminB1 were maintained by blocking the proteasome in both shGFP and shMT1-MMP expressing cells (Fig 1C), indicating laminB1 degradation is through the proteasome as was previously described [30].

Since we have shown that MT1-MMP protects against DNA damage through ECM processing and activation of integrinβ1/FAK [8], we asked if ECM remodeling and integrinβ1/FAK pathway activation might mediate MT1-MMP dependent stability of laminB1. First, we found that cells embedded in hydrogel matrixes resistant to degradation by MT1-MMP [8] showed a marked decrease in laminB1 protein (Fig 1D), concomitant to decreased phospho-FAK^Y397^, indicating the processing of the ECM is a requirement in maintaining the levels of laminB1, possibly via FAK signaling. Indeed, decreased FAK activity that follows MT1-MMP depletion was associated with reduced laminB1 (Fig 1E). Importantly, cells treated with an FAK kinase inhibitor (VS4718) [19–21] showed increased laminB1 degradation (Fig 1F and 1G), supporting a role of FAK activity in laminB1 stability.

Since FAK has been shown to phosphorylate and stabilize several proteins including S-phase Kinase-associated Protein-2 (Skp-2) [31]; and PTEN [32], we then tested whether FAK and laminB1 could physically interact, a prerequisite for enzyme-substrate activity. Co-imunoprecipitation using either laminB1 (Fig 1H) or FAK (Fig 1I) as bait showed the two proteins form a complex. Additionally, FAK and laminB1 were found to partly co-localized at the nuclear rim in cells expressing shGFP, however subcellular localization of FAK was perturbed upon MT1-MMP depletion (S1 Fig) Together, these data support the notion that laminB1 stability is positively regulated by integrinβ1/FAK signaling pathway triggered by MT1-MMP dependent ECM processing.

### LaminB1 regulates DNA damage and DNA replication stress triggered by MT1-MMP inhibition

**Fig 2:**
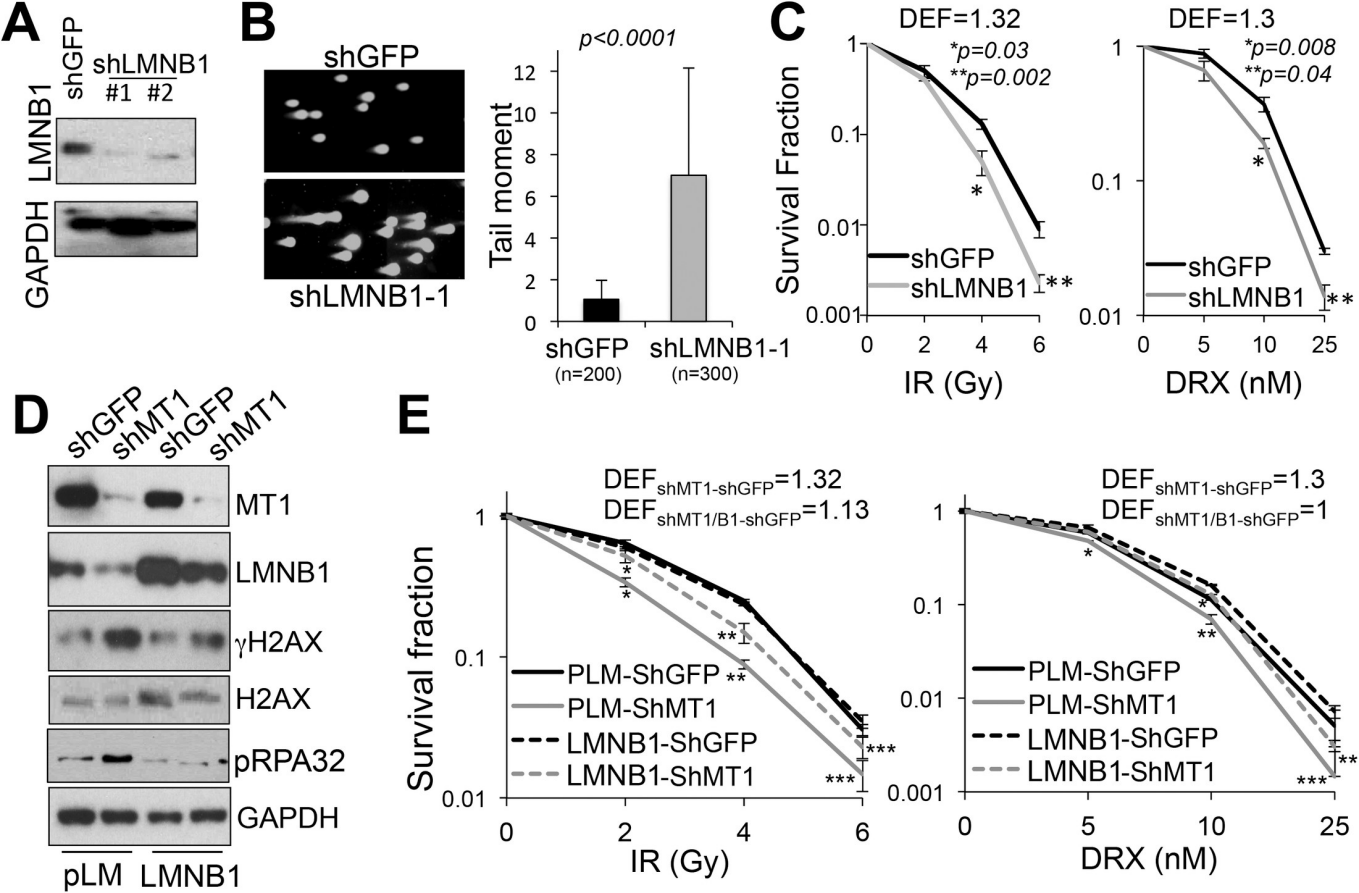
LaminB1 inhibition promotes DSBs and sensitizes to IR and doxorubixn. **A)** Western blotting of total lysates of MDA-MB-231 cells expressing shGFP or two shRNAs against laminB1. **B)** Representative picture of a neutral comet assay of MDA-MB-231 cells expressing shGFP or sh-laminB1-1. Tail moment was calculated on at least 50 comets per condition, using Comet Score (TriTek). **C)** Clonogenic assay of MDA-MB-231 cells expressing shGFP or sh-laminB1-1 treated with IR and DRX for 15 days. DEF (Dose Enhancement Factor) is shown. DEF was determined by the ratio between the radiation or DRX dose alone at 0.1 survival fraction versus radiation or DRX + shLMNB1 at the same survival fraction (DEF>1 = sensitizer; DEF<1 = radioprotector). Significant difference between shGFP and shLMNB1 was observed at 4 and 6 Gy IR and 10 and 25 nM DRX, Student’s *t* test. **D)** MT1-MMP, laminB1, γH2AX, total H2AX, phospho-RPA32 and GAPDH levels in cells expressing shGFP or shMT1-MMP and transduced with exogenous laminB1. **E)** Clonogenic assay of the cells in D treated with IR and DRX for 15 days. DEF is shown. Left panel: *^,^**^,^***p<0.001, significant difference between shGFP and shMT1-MMP was observed at 2, 4, 6 Gy IR; *^,^**p<0.001 and ***p<0.01 significant difference between shGFP and shMT1-MMP in which laminB1 was restored was observed at 2, 4, 6 Gy IR. Right panel: *^,^**^,^***p<0.01 significant difference between shGFP and shMT1-MMP was observed at 5, 10, 25 nM DRX, Student’s *t* test; *^,^**p<0.01 significant difference between shGFP and shMT1-MMP in which laminB1 was restored was observed at 5, 10, 25 nM DRX, Student’s *t* test.

After establishing a connection between MT1-MMP dependent ECM processing and laminB1 stability, we next asked whether laminB1 inhibition could directly sensitize breast cancer cells to radiation and chemotherapy similarly to depletion of MT1-MMP. To this end, laminB1 was inhibited by specific shRNAs (Fig 2A), and then a neutral comet assay was performed to quantify DSBs (Fig 2B). Cells depleted of laminB1 showed a 7-fold increase in endogenous DSBs. When the cells were then treated with increasing doses of either IR or doxorubicin, we observed sensitization to both forms of genotoxic treatments (Fig 2C). On the other hand, restoration of laminB1 in shMT1-MMP cells decreased the total level of phosphorylated H2AX (γH2AX) as well as the number of γH2AX nuclear foci, indicating a reduction in endogenous DNA damage (Figs 2D and S2), and partly rescued the resistance to both IR and doxorubicin (Fig 2E).

**Fig 3:**
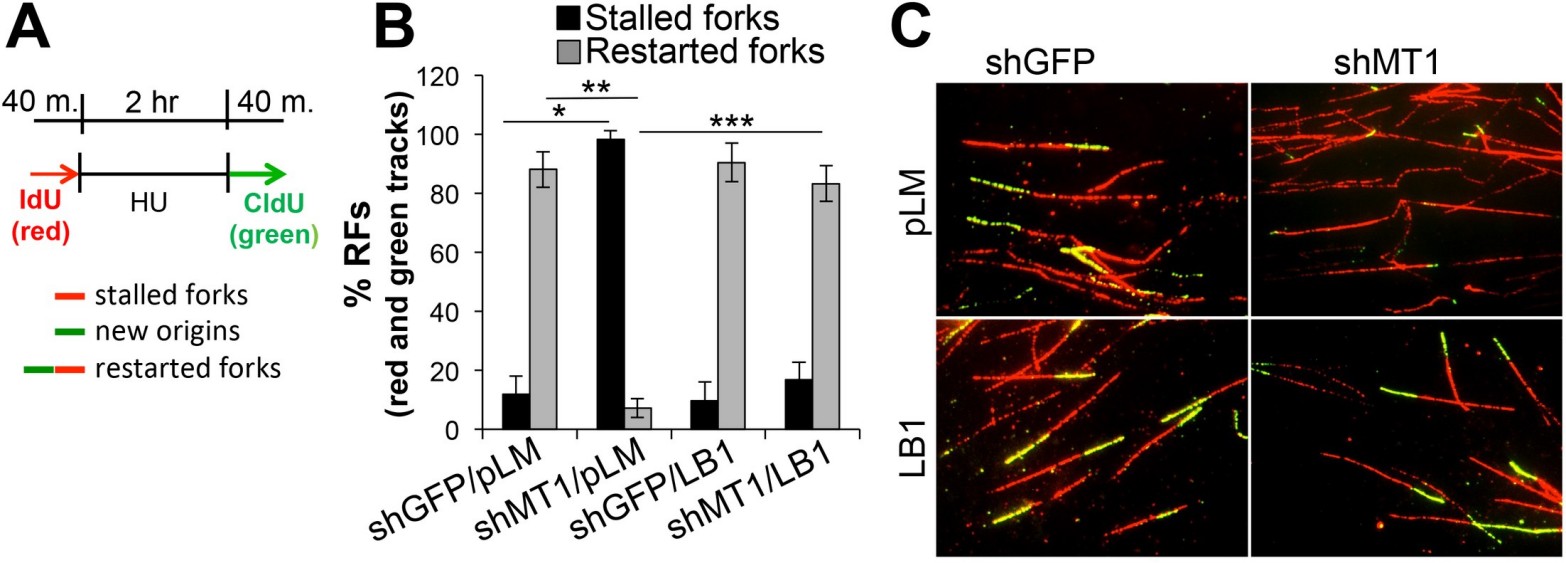
LaminB1 rescues stalling of replication forks caused by MT1-MMP inhibition. **A)** Schematic of experiment. Cells are pulsed with the nucleotide analogue IdU (red), followed by depletion of the nucleotide pool with hydroxyurea, and chase with a second nucleotide analogue (green). Red only tracks: Stalled forks; red and green tracks: Restarted forks; green only tracks: New origins. **B)** % of replication forks (% RFs) was calculated by counting at least 200 red and green tracks for each condition. *,**,***p<0.0001, Student’s *t* test. **C)** Representative CldU (green) and IdU (red) stained DNA fibers.

Restoration of laminB1 also counteracted the phosphorylation of RPA32, a marker of DNA replication stress (Fig 2D). Hence, based on this evidence, and the fact that we have shown MT1-MMP inhibition causes stalling and collapse of replication forks [8], we conducted a DNA fiber assay to determine if laminB1 could protect from replication stress. To do so, cells were first labeled with the nucleotide analogue IdU followed by hydroxyurea to deplete the nucleotide pool, and then were labeled with a second nucleotide analogue CldU to determine if cells can recover DNA synthesis. Indeed, over-expression of laminB1 was sufficient to restore DNA replication as indicated by the increase in restarted forks (rend and green tracks) to control levels in cells depleted of MT1-MMP (Fig 3A–3C). Together, these data support a role of laminB1 in DNA damage responses and RF stability downstream of MT1-MMP.

### LaminB1 is present at replication forks (RF) where it may contribute to RF stability

**Fig 4:**
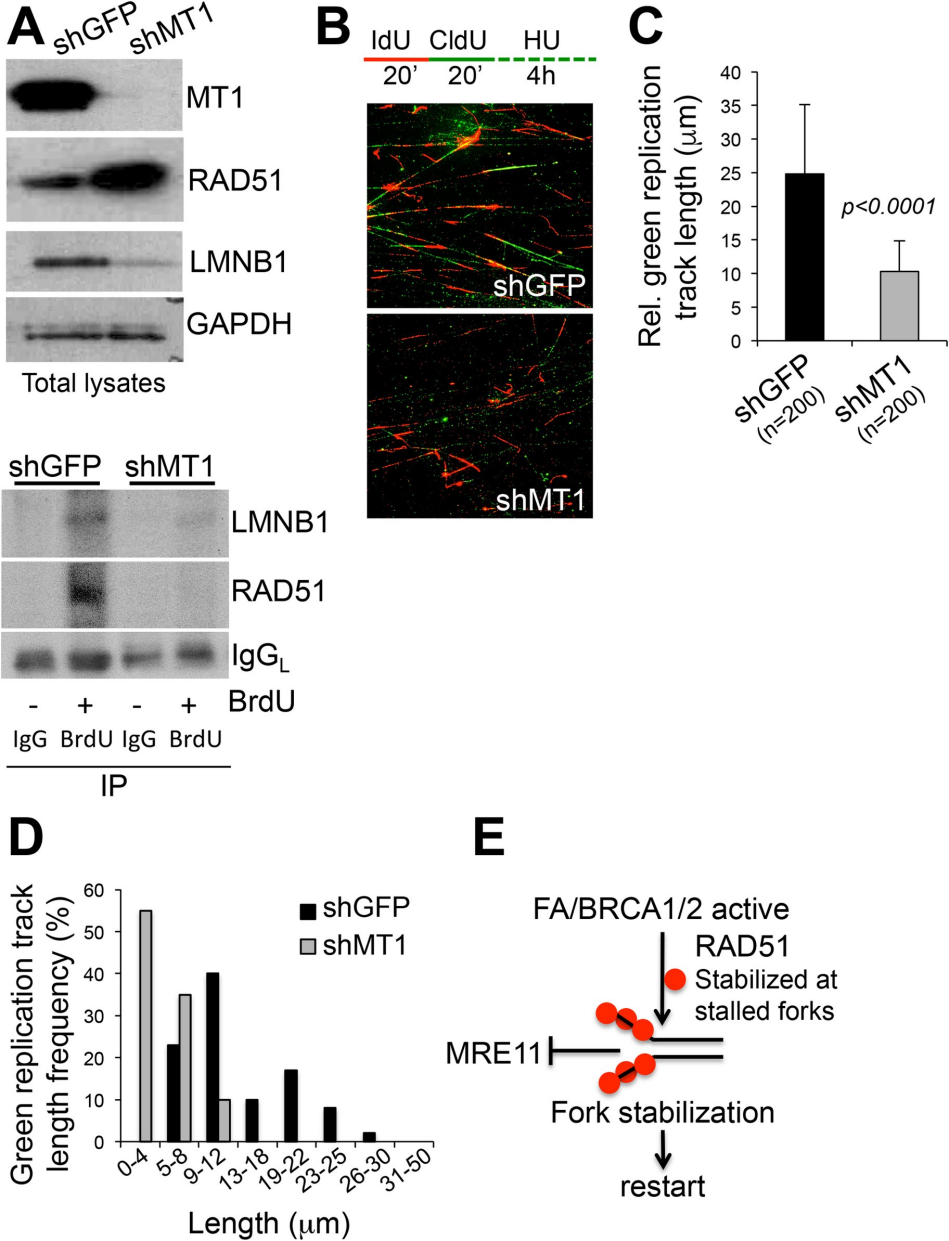
MT1-MMP regulates laminB1 association to RFs and RF degradation. **A)** Upper panel: Western blotting of total lysates of MDa-MB-231 cells expressing shGFP or shMT1-MMP. Levels of LaminB1 (LMNB1) and of the RF protection factor RAD51 are shown. GAPDH was used as loading control. Bottom panel: Cells in A were labeled with BrdU (+) or left unlabeled (-), then BrdU immunoprecipitation was performed. An IgG antibody was used to immunoprecipitate unlabeled samples. IgG light chain (IgG_L_) was used as loading control. Western blotting shows levels of laminB1 and RAD51 bound to replication forks. **B)** Cells expressing shGFP or shMT1-MMP were pulsed with IdU/CldU following chase with Hydroxyurea (representative pictures of shGFP and shMT1-MMP expressing cells). **C)** Average length of replication green (CldU) track after Hydroxyurea chase (μm). The length of at least 200 tracks was measured using ImageJ. **D)** % Distribution of replication tracks after Hydroxyurea chase based on track relative length. The length of at least 200 tracks was measured using ImageJ. **E)** Fanconi Anemia (FA) pathway with BRCA2 and BRCA1 operate to allow stabilization of RAD51 to stalled RFs, which then counteract the nuclease activity of MRE11, thus stabilizing the fork and allowing restart.

The mechanism by which laminB1 promotes RF stability is unknown. We have hypothesized that laminB1 might be present at RFs where it contributes to RF stability. Evidence supporting this possibility comes from a work by Moir *et al*, in which laminB1 was found to co-localize with proliferating cell nuclear antigen (PCNA) at sites of DNA synthesis in late S phase [33]. Additionally, lamins have been shown to be required for the proper positioning of PCNA on chromatin during the elongation phase of DNA replication [34]. Hence, we tested whether laminB1 was associated with RFs in our cell system. Cells were labeled with the nucleotide analogue BrdU (+) or left unlabeled (-), followed by DNA-protein crosslinking and BrdU immuno-precipitation with a specific antibody. BrdU (-) samples were immunoprecipitated with a control IgG. Interestingly, laminB1 was found at sites of BrdU incorporation, indicating laminB1 is indeed associated with RFs (Fig 4A, bottom panel). Inhibition of MT1-MMP, which causes degradation of laminB1, was associated with reduced laminB1 at RFs. This phenomenon coincided with accelerated degradation of RFs, as shown in Fig 4B–4D, in a pulse/chase DNA fiber assay. Here, the speed of RF degradation is measured by pulsing cells with IdU and CldU, followed by a chase with hydroxyurea to cause nucleotide pool depletion. The average length of the green tracts (Fig 4C) and the distribution of the green tracts according to their length (Fig 4D) show that the overall length of the CIdU labeled tracts are shorter supporting accelerated degradation. We therefore hypothesized that laminB1 may work as a scaffold protein to allow the positioning of protection factors at RFs, thus preventing RF degradation. Key factors involved in RF protection are the Fanconi Anemia Pathway and BRCA1/2, which cooperate to promote the stability of stalled replication forks by protecting them against degradation by MRE11 nuclease through stabilization of RAD51 filaments at stalled forks (Fig 4E) [35,36]. Of note, it has been previously shown that laminB1 stabilizes RAD51 and that stable RAD51 then facilitates the repair of DSBs induced by IR [37]. We tested whether MT1-MMP depletion affected the levels of RAD51. Interestingly, upon MT1-MMP inhibition, while laminB1 decreased, total RAD51 increased, likely in response to the increased DNA damage (Fig 4A, top panel). However, RAD51 levels paradoxically decreased at RFs (Fig 4A, bottom panel), likely explaining the observed higher degradation of RFs. To further elucidate the relationship between RAD51 and RFs, we performed co-localization of RAD51 and BrdU by immunofluorescence in BrdU labeled cells expressing either shGFP, shMT1-MMP or shlaminB1 (shLMNB1) (S3 and S4 Figs). We found that RAD51 and BrdU co-localize in control (shGFP) cells but not in shMT1-MMP and shLMNB1, where RFs tend to localize at the periphery of the nucleus and RAD51 more at the center, with few or no points of co-localization. This pattern resembles cells in which prolonged stalling of RFs causes them to relocate at the nuclear periphery, in contact with the Nuclear Pore Complex, as reviewed in Whalen et al [38]. Hence, it is possible that chronic depletion of MT1-MMP or laminB1 caused by stable RNA silencing, may lead to a similar pattern. While the underlying mechanism(s) of such behavior remain to be further investigated, the data presented thus far, support a novel role of laminB1 in RF stability through the proper loading of RAD51 to RFs, a prerequisite for the repair of RFs [39].

## Conclusions

Here we establish a novel mechanism of replication fork protection whereby MT1-MMP, via cleavage of the ECM promotes laminB1 stability though the activation of integrinB1/FAK signaling. LaminB1 then likely functions as a scaffold for RF protection factors such as RAD51, to facilitate their positioning to prevent fork degradation. Thus, MT1-MMP mediates a novel cell/ECM mechanism of DNA repair in triple negative breast cancer cells. A role of MT1-MMP in resistance to genotoxic stresses has been observed also in glioblastoma multiforme, where MT1-MMP depletion sensitizes to radiation and temozolomide [40]; and in MT1-MMP knock-out mouse fibroblasts, where lack of MT1-MMP causes premature senescence and accumulation of DNA damage [41]. Whether other MMPs may cause a similar phenotype remains to be established. For example, MMP2 inhibition has been shown to sensitize lung cancer cells and MCF7 breast cancer cells to radiation, but to protect human fibroblasts from the same stress [42,43]. In a recent work by Walter et al [44], MMP9 expression was shown to exert a protective role in colitis-associated cancer (CAC), where MMP9 protected against ROS and reduced DNA damage, thus counteracting CAC. This suggests different MMPs may either be radio-protective or radio-sensitizing depending on the context in which they operate.

While we have established a link between FAK and laminB1 in promoting the stability of the latter, it still remains to be determined how and on what site(s) FAK phosphorylates laminB1 and whether this phosphorylation is necessary and sufficient to prevent its proteasome-dependent degradation. Finally, although these findings were generated in a triple negative breast cancer model, used as a prototypic system undergoing radiation therapy, a majority of aggressive solid tumors express high levels of MT1-MMP thus, it is likely this novel mechanism of DNA repair may be present in other cancer types where it contributes to radioresistance.

## Supporting information

S1 Fig FAK and laminB1 co-localize at the nuclear rim in shGFP expressing cells. % co-localization: 3% +/- 0.07 (ImageJ). Cells transduced with an shRNA against MT1-MMP express low laminB1. % co-localization: 0% (ImageJ). Images were taken at 60X magnification. Each color channel was maintained at the same intensity for both shGFP and shMT1 cells.
(PDF)

S2 Fig A) % of cells with more than 10 γH2AX foci per nucleus. at least 50 nuclei were counted in 5 fields per slide, in triplicate, for each condition. B) representative pictures of nuclei (DAPI stained, blue) with γH2AX foci (red) of the cells in A (magnification: 60X). Cells were infected with shRNAs against GFP (shGFP) or MT1-MMP (shMT1) following by stable expression of wild type laminB1 (LMNB1) or an empty lentiviral vector (pLM).
(PDF)

S3 Fig A) LaminB1 (red) and MT1-MMP (green) expression in MDA-MB-231 expressing shGFP or shMT1-MMP. B) BrdU (green) and RAD51 (red) nuclear localization of the cells in A. % colocalization: shGFP: 24% +/- 3; shMT1-MMP: 4.25% +/- 0.8 (ImageJ). Magnification: 60X for both A and B.
(PDF)

S4 Fig A) LaminB1 (green) and DAPI(blue) in MDA-MB-231 expressing shGFP or shLaminB1 (shLMNB1). B) BrdU (green) and RAD51 (red) nuclear localization of the cells in A. Magnification: 40X (A); 60X (B).
(PDF)

S1 Raw images (PDF)
