# Supplementary material for: MT1-MMP-dependent ECM processing regulates laminB1 stability and mediates replication fork restart
Source: PLoS One. 2021 Jul 8;16(7):e0253062. doi: 10.1371/journal.pone.0253062 (PMC8266045; doi:10.1371/journal.pone.0253062)
Supplement: S1 Fig — % co-localization: 3% +/- 0.07 (ImageJ). Cells transduced with an shRNA against MT1-MMP express low laminB1. % co-localization: 0% (ImageJ). Images were taken at 60X magnification. Each color channel was maintained at the same intensity for both shGFP and shMT1 cells. (PDF) [file pone.0253062.s001.pdf]

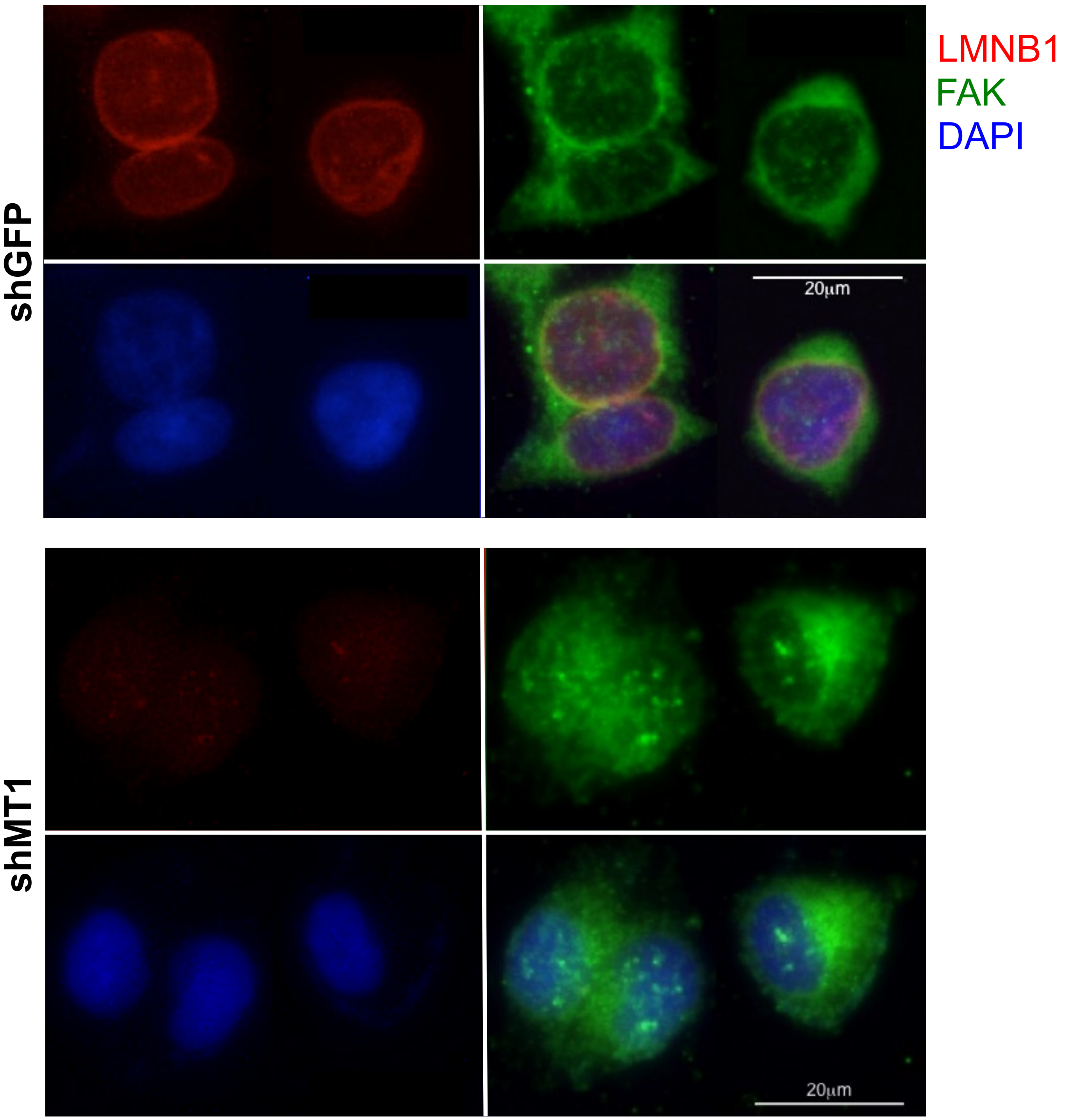

Suppl. Figure 1: FAK and laminB1 co-localize at the nuclear rim in shGFP expressing cells. % co-localization: 3% +/- 0.07 (ImageJ). Cells transduced with an shRNA against MT1-MMP express low laminB1. % co-localization: 0% (ImageJ). Images were taken at 60X magnification. Each color channel was maintained at the same intensity for both shGFP and shMT1 cells.
