# Supplementary material for: MT1-MMP-dependent ECM processing regulates laminB1 stability and mediates replication fork restart
Source: PLoS One. 2021 Jul 8;16(7):e0253062. doi: 10.1371/journal.pone.0253062 (PMC8266045; doi:10.1371/journal.pone.0253062)
Supplement: S2 Fig — A) % of cells with more than 10 γH2AX foci per nucleus. at least 50 nuclei were counted in 5 fields per slide, in triplicate, for each condition. B) representative pictures of nuclei (DAPI stained, blue) with γH2AX foci (red) of the cells in A (magnification: 60X). Cells were infected with shRNAs against GFP (shGFP) or MT1-MMP (shMT1) following by stable expression of wild type laminB1 (LMNB1) or an empty lentiviral vector (pLM). (PDF) [file pone.0253062.s002.pdf]

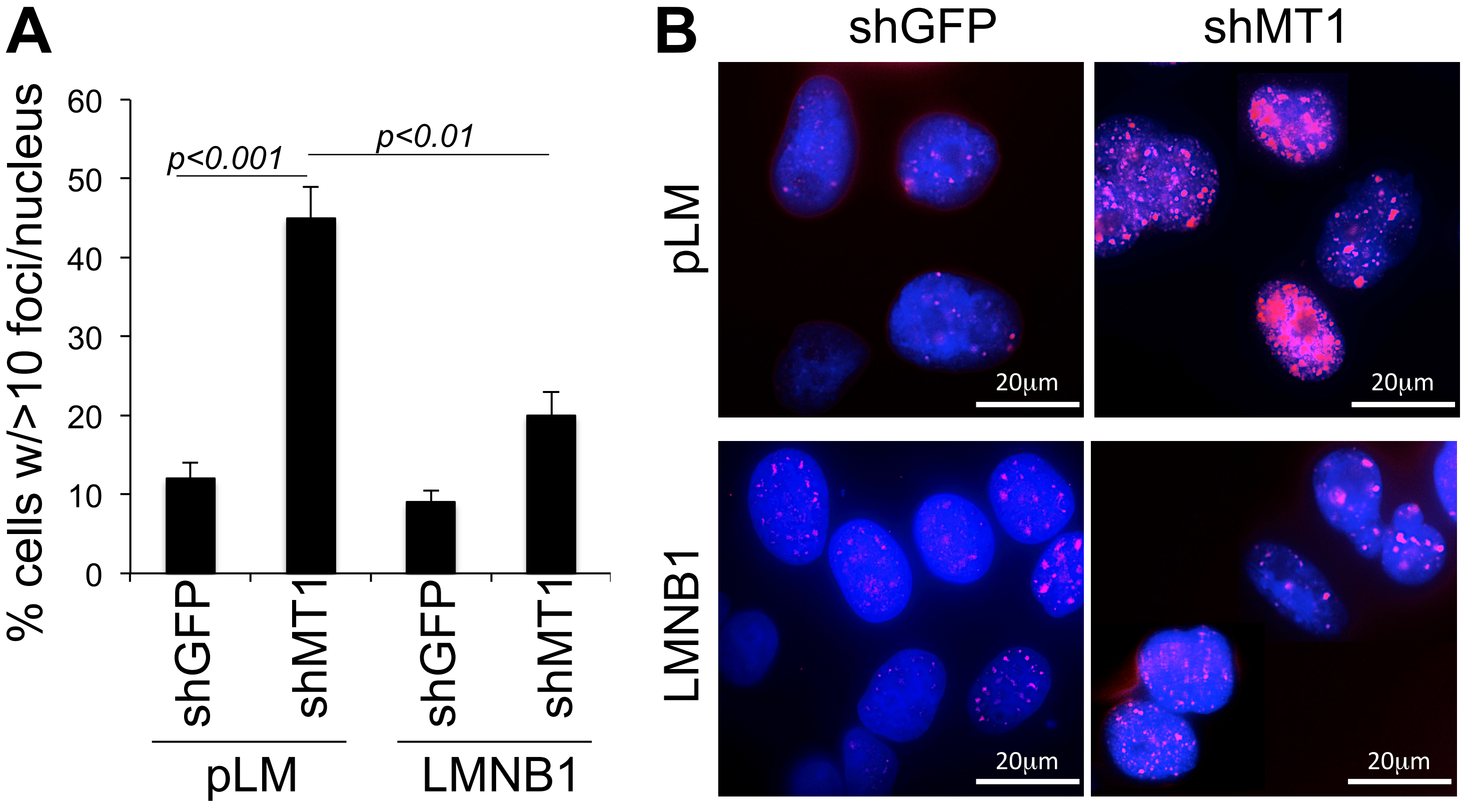

Suppl. Figure 2: A) % of cells with more than 10 γH2AX foci per nucleus. at least 50 nuclei were counted in 5 fields per slide, in triplicate, for each condition. **B)** representative pictures of nuclei (DAPI stained, blue) with γH2AX foci (red) of the cells in A (magnification: 60X). Cells were infected with shRNAs against GFP (shGFP) or MT1-MMP (shMT1) following by stable expression of wild type laminB1 (LMNB1) or an empty lentiviral vector (pLM).
