# Supplementary material for: MT1-MMP-dependent ECM processing regulates laminB1 stability and mediates replication fork restart
Source: PLoS One. 2021 Jul 8;16(7):e0253062. doi: 10.1371/journal.pone.0253062 (PMC8266045; doi:10.1371/journal.pone.0253062)
Supplement: S3 Fig — A) LaminB1 (red) and MT1-MMP (green) expression in MDA-MB-231 expressing shGFP or shMT1-MMP. B) BrdU (green) and RAD51 (red) nuclear localization of the cells in A. % colocalization: shGFP: 24% +/- 3; shMT1-MMP: 4.25% +/- 0.8 (ImageJ). Magnification: 60X for both A and B. (PDF) [file pone.0253062.s003.pdf]

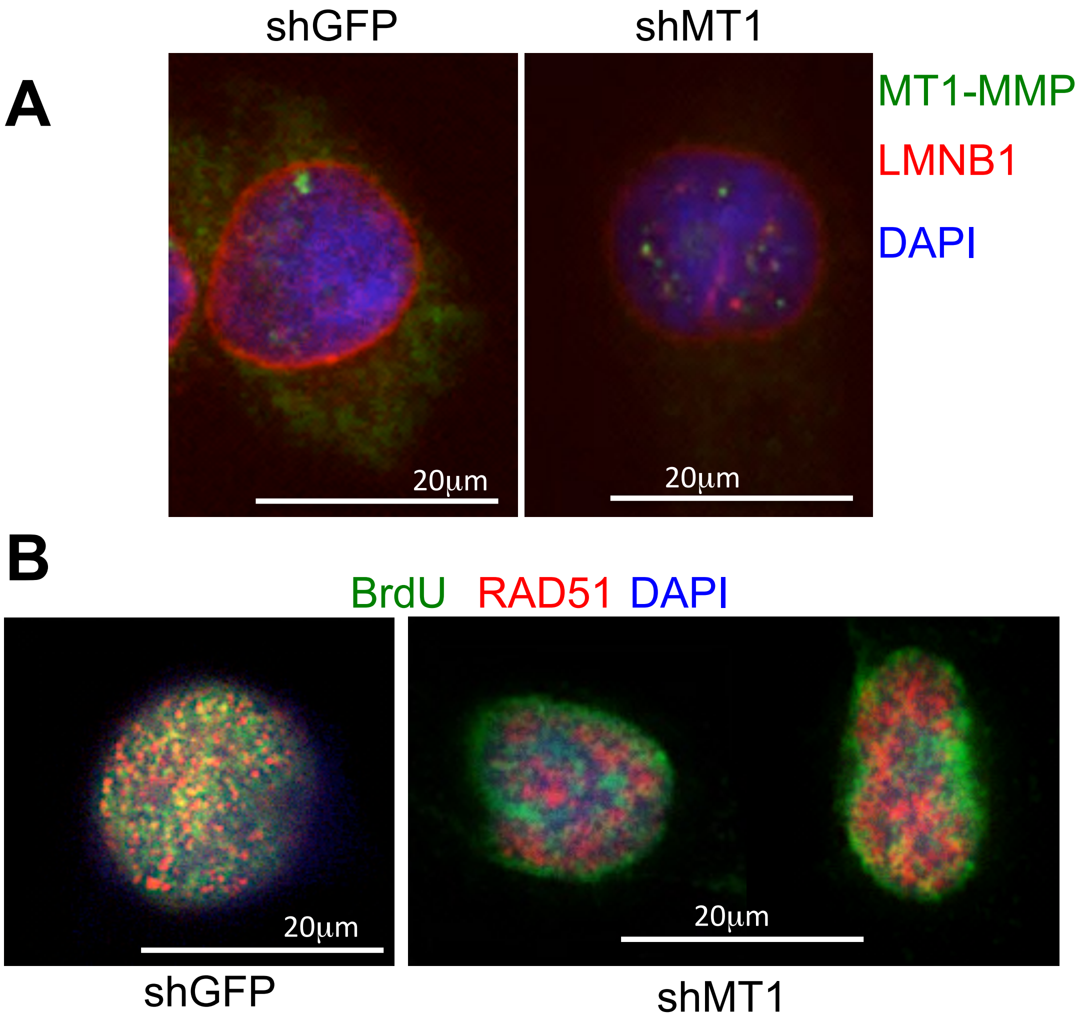

Suppl. Figure 3: A) LaminB1 (red) and MT1-MMP (green) expression in MDA-MB-231 expressing shGFP or shMT1-MMP. **B)** BrdU (green) and RAD51 (red) nuclear localization of the cells in A. % co-localization: shGFP: 24% +/- 3; shMT1-MMP: 4.25% +/- 0.8 (ImageJ). Magnification: 60X for both A and B.
