# Supplementary material for: MT1-MMP-dependent ECM processing regulates laminB1 stability and mediates replication fork restart
Source: PLoS One. 2021 Jul 8;16(7):e0253062. doi: 10.1371/journal.pone.0253062 (PMC8266045; doi:10.1371/journal.pone.0253062)
Supplement: S4 Fig — A) LaminB1 (green) and DAPI(blue) in MDA-MB-231 expressing shGFP or shLaminB1 (shLMNB1). B) BrdU (green) and RAD51 (red) nuclear localization of the cells in A. Magnification: 40X (A); 60X (B). (PDF) [file pone.0253062.s004.pdf]

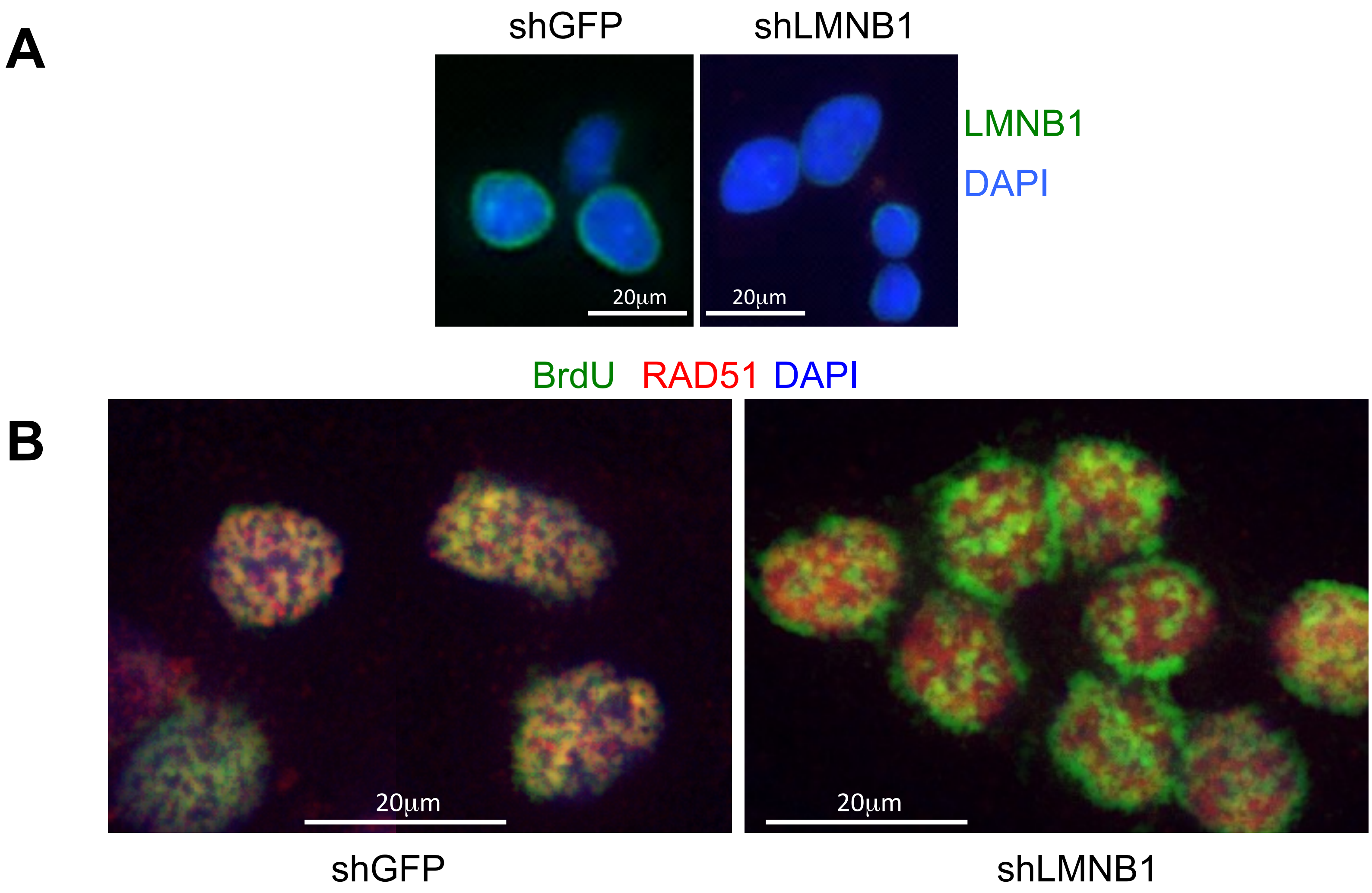

Suppl. Figure 4: A) LaminB1 (green) and DAPI(blue) in MDA-MB-231 expressing shGFP or shLaminB1 (shLMNB1). **B)** BrdU (green) and RAD51 (red) nuclear localization of the cells in A. Magnification: 40X (A); 60X (B).
