# Supplementary material for: MT1-MMP-dependent ECM processing regulates laminB1 stability and mediates replication fork restart
Source: PLoS One. 2021 Jul 8;16(7):e0253062. doi: 10.1371/journal.pone.0253062 (PMC8266045; doi:10.1371/journal.pone.0253062)

Fig. 1B

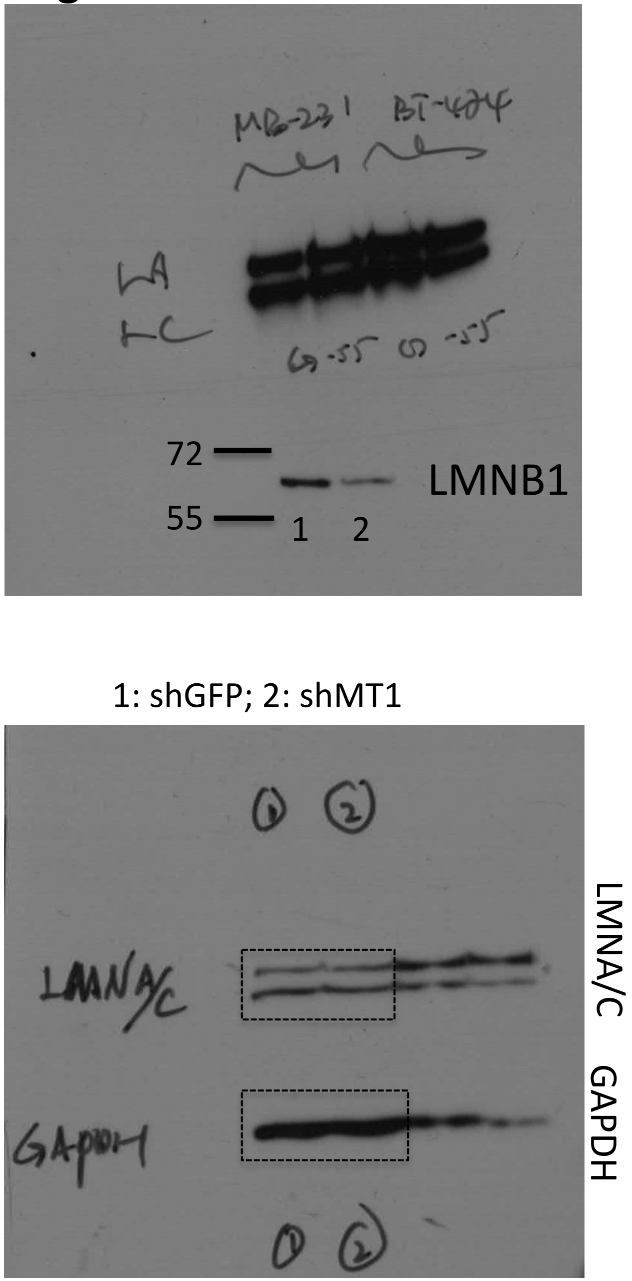
72
55
LMNB1
1 2
1: shGFP; 2: shMT1
LMNA/C
GAPDH

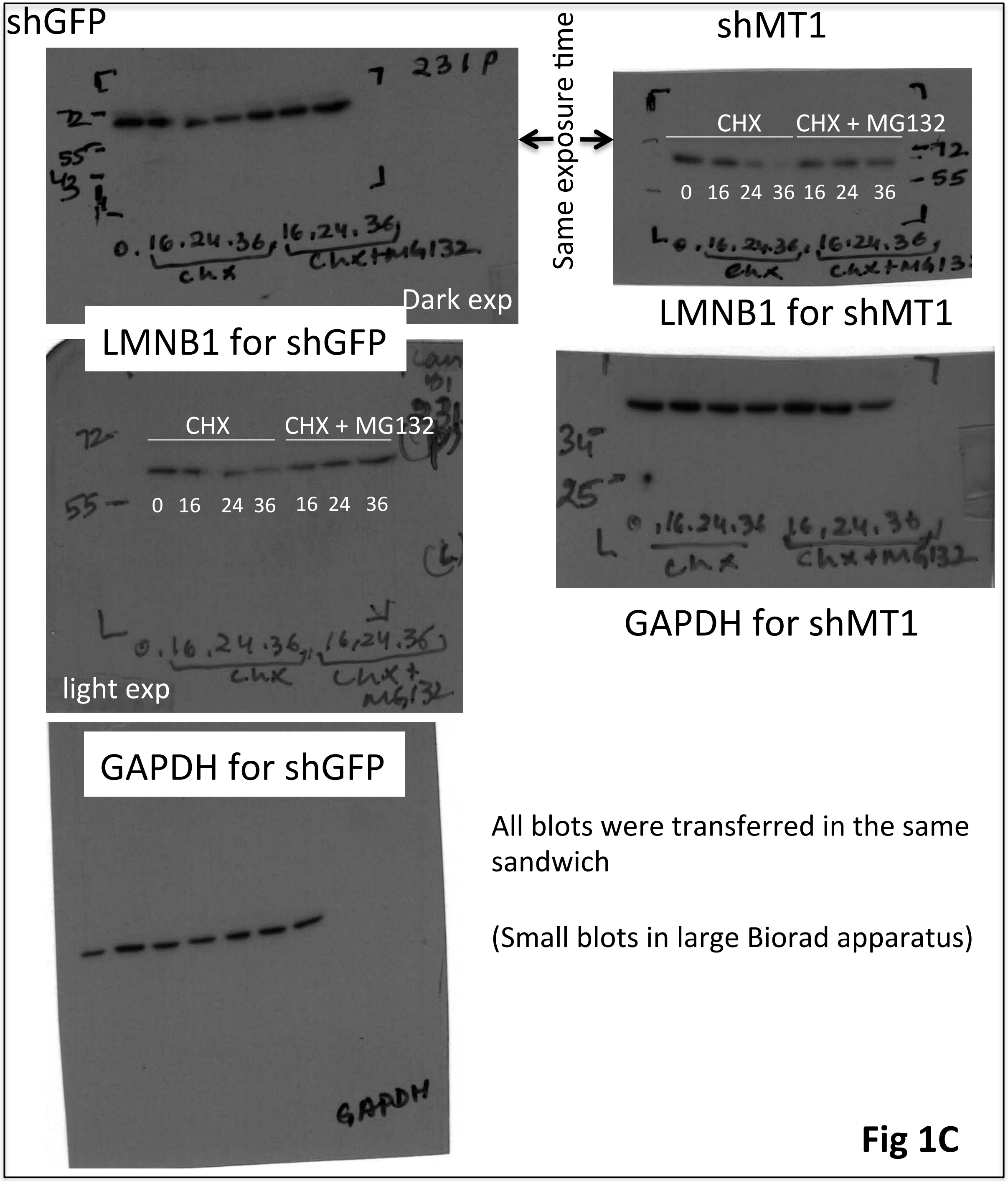
shGFP
shMT1
Same exposure time
CHX
CHX + MG132
0 16 24 36 16 24 36
Dark exp
LMNB1 for shMT1
LMNB1 for shGFP
light exp
GAPDH for shMT1
GAPDH for shGFP
All blots were transferred in the same sandwich
(Small blots in large Biorad apparatus)

Fig 1C

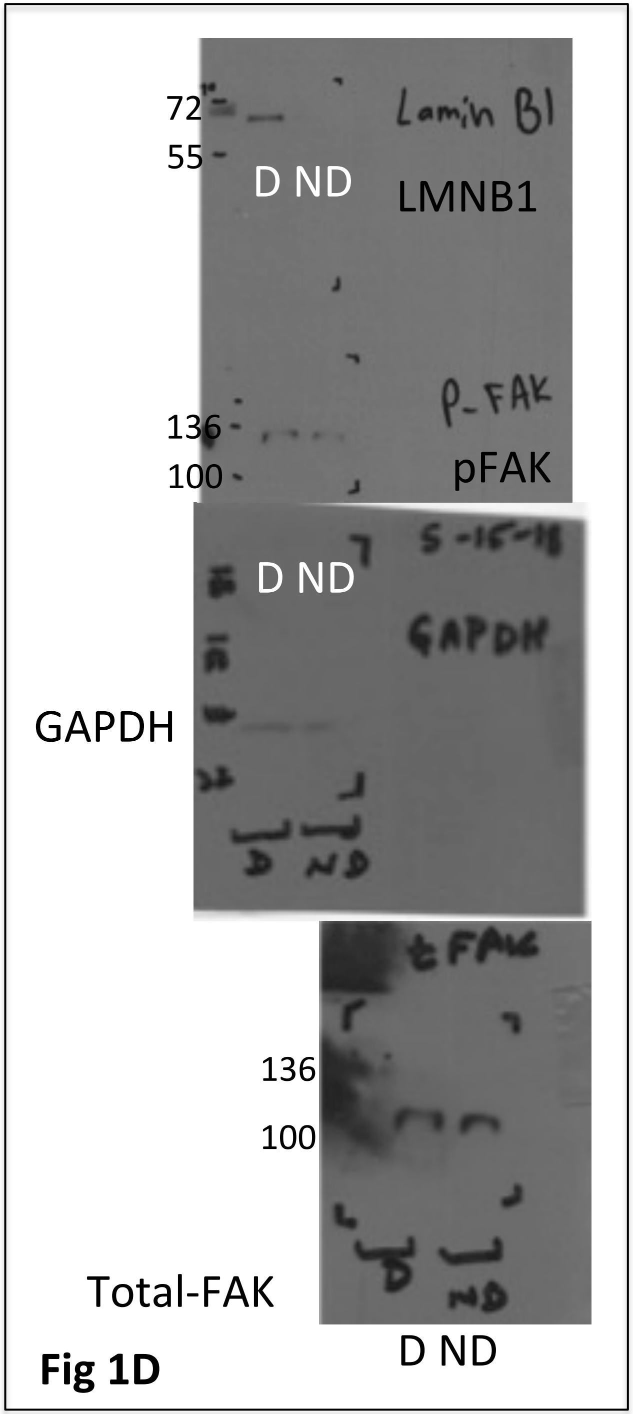
72
55
D ND
LMNB1
136
100
pFAK
D ND
GAPDH
136
100
Total-FAK
D ND

Fig 1D

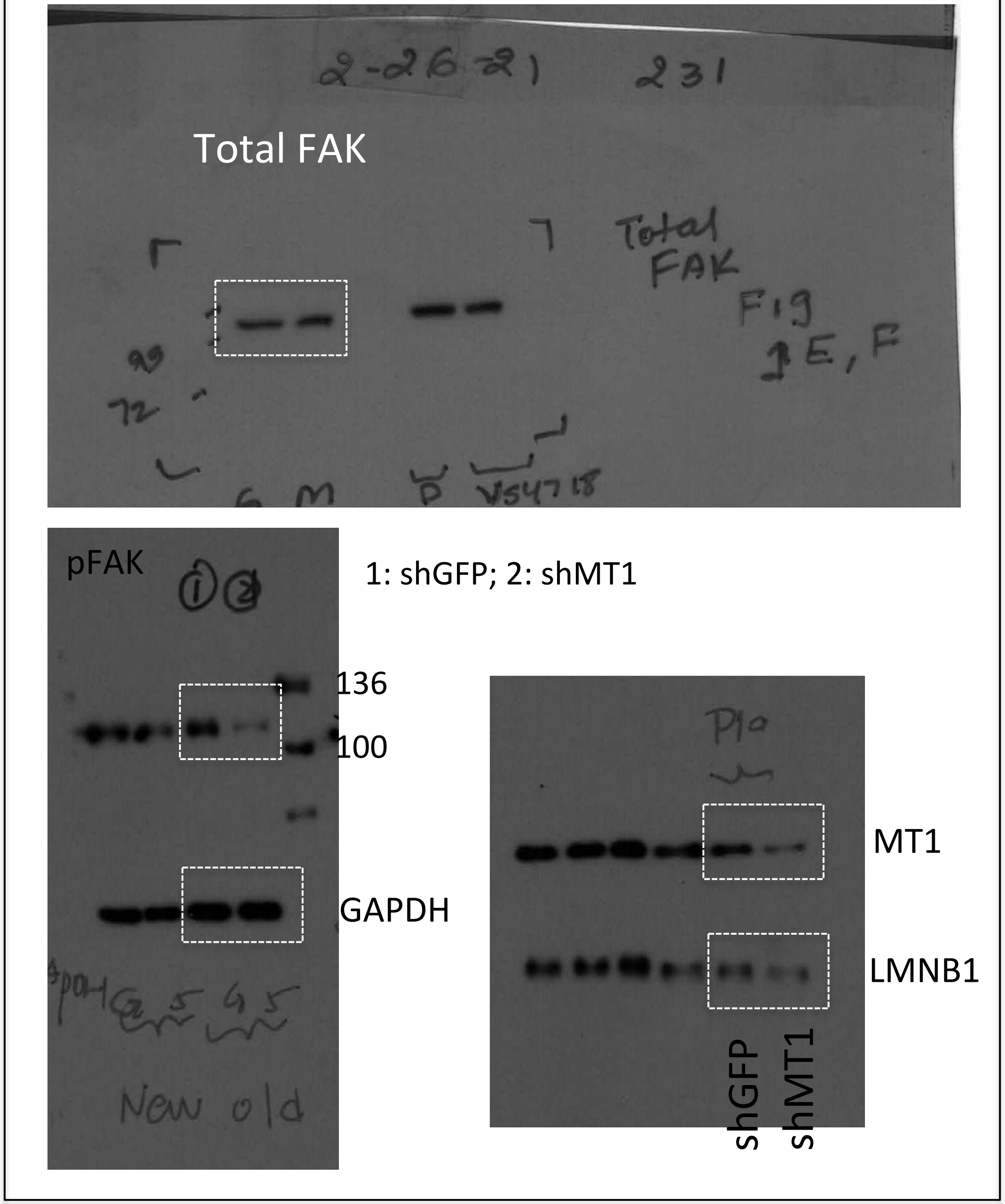
Total FAK
pFAK
1: shGFP; 2: shMT1
136
100
GAPDH
MT1
LMNB1
shGFP
shMT1

Fig. 1E

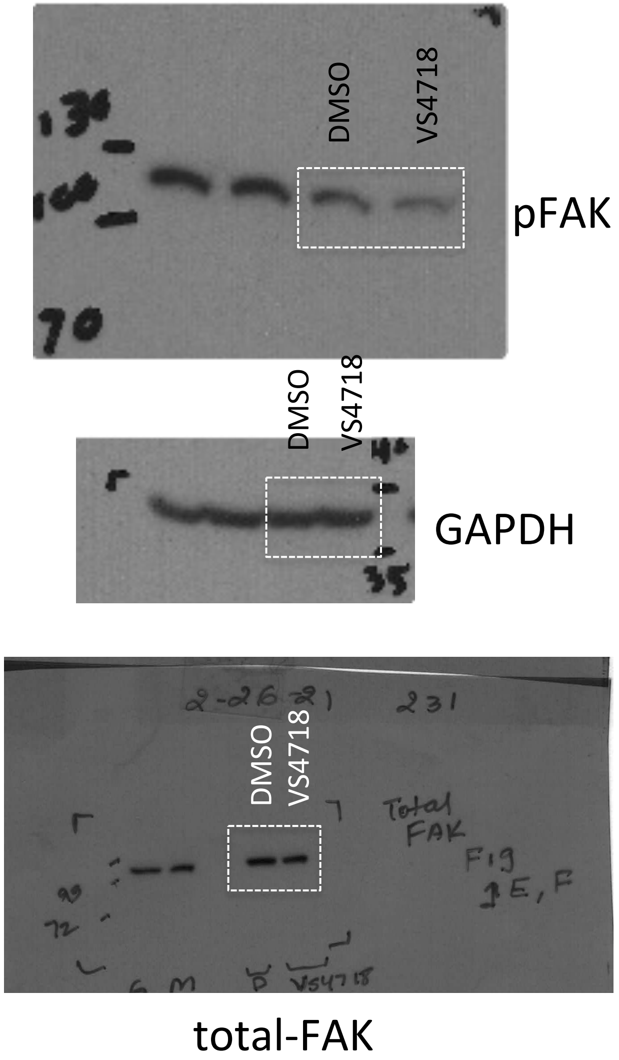
DMSO
VS4718
pFAK
DMSO
VS4718
GAPDH
DMSO
VS4718
total-FAK

Fig. 1F

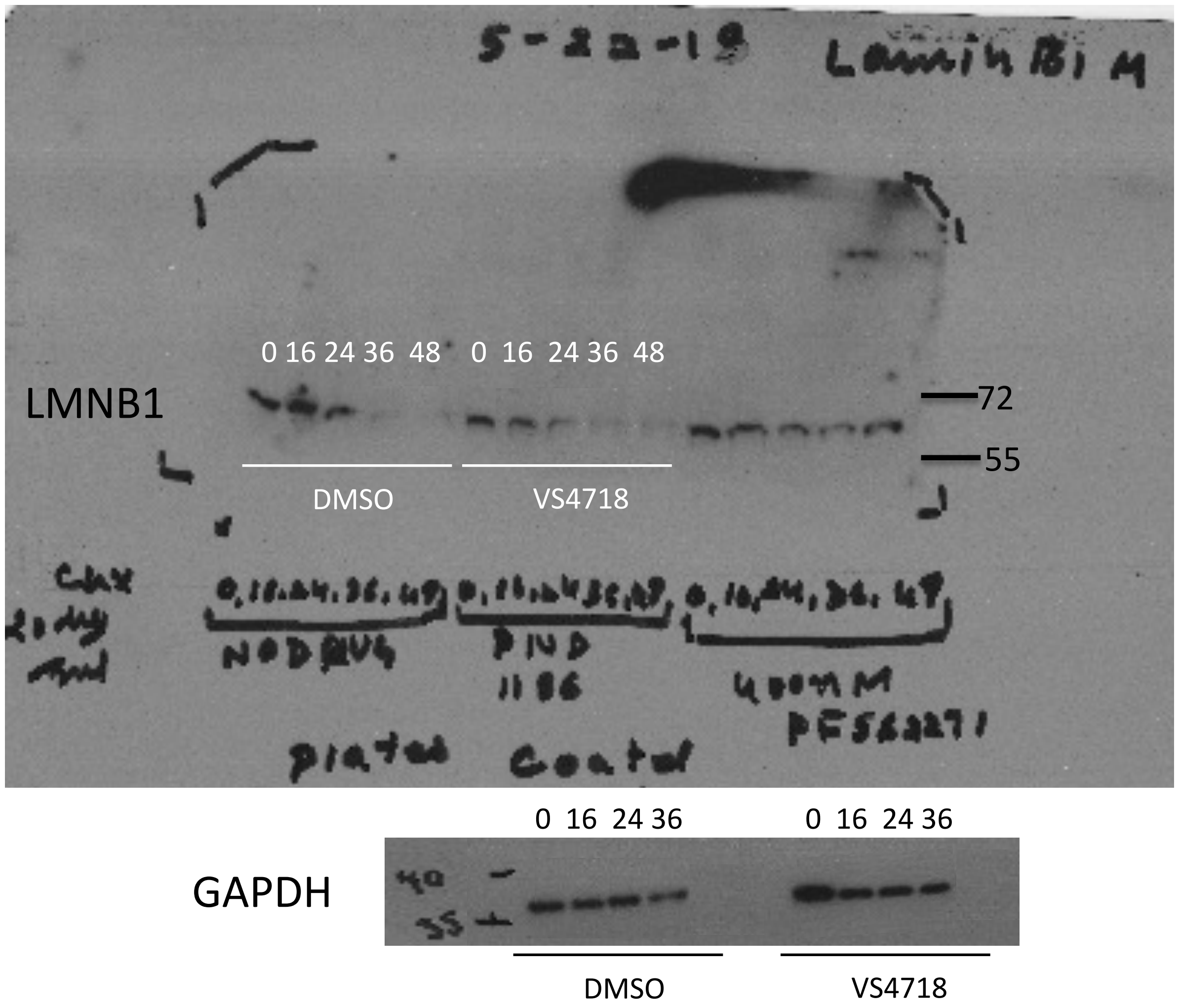
0 16 24 36 48 0 16 24 36 48
LMNB1
72
55
DMSO
VS4718
0 16 24 36
0 16 24 36
GAPDH
DMSO
VS4718

Fig. 1G

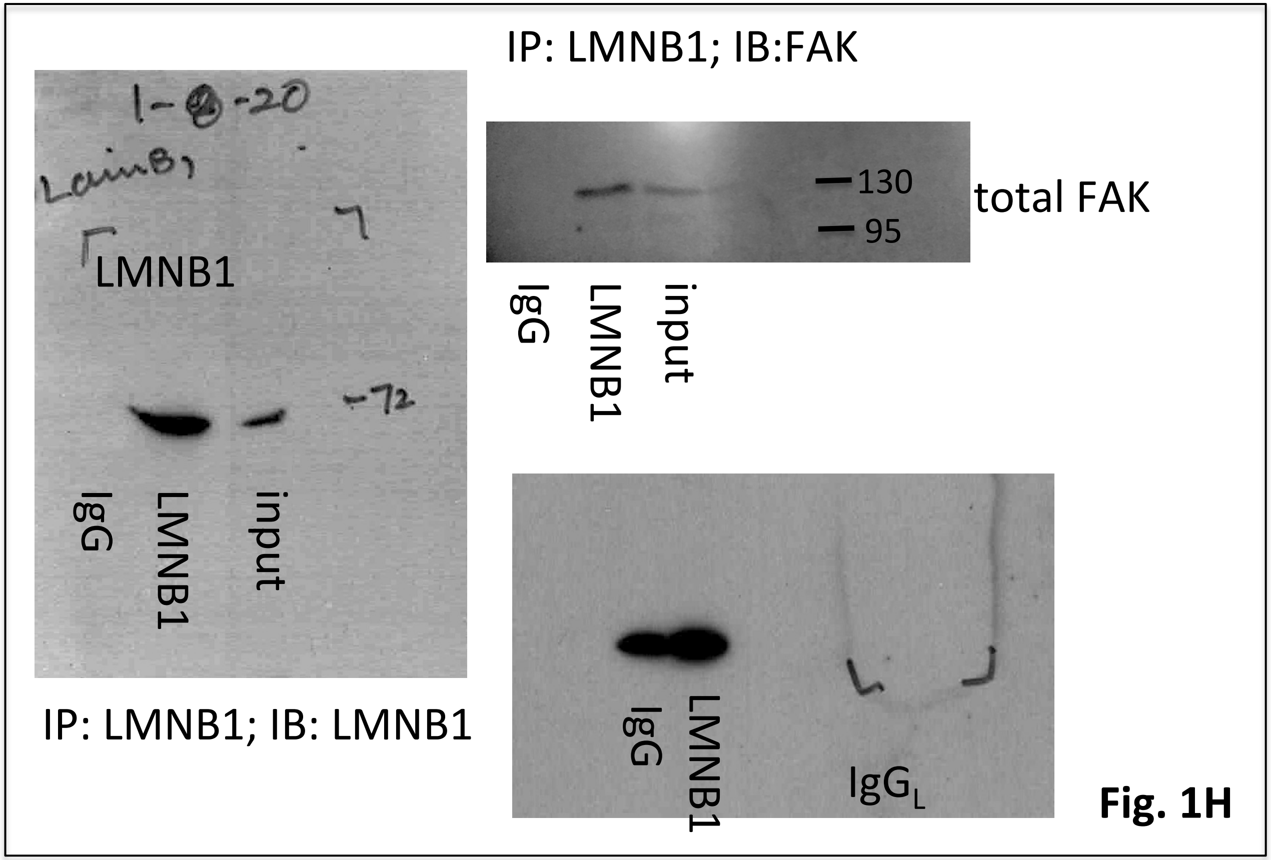
IP: LMNB1; IB:FAK
LMNB1
IgG
LMNB1
input
130
95
total FAK
IgG
LMNB1
input
IgG
LMNB1
IgG_L
IP: LMNB1; IB: LMNB1

Fig. 1H

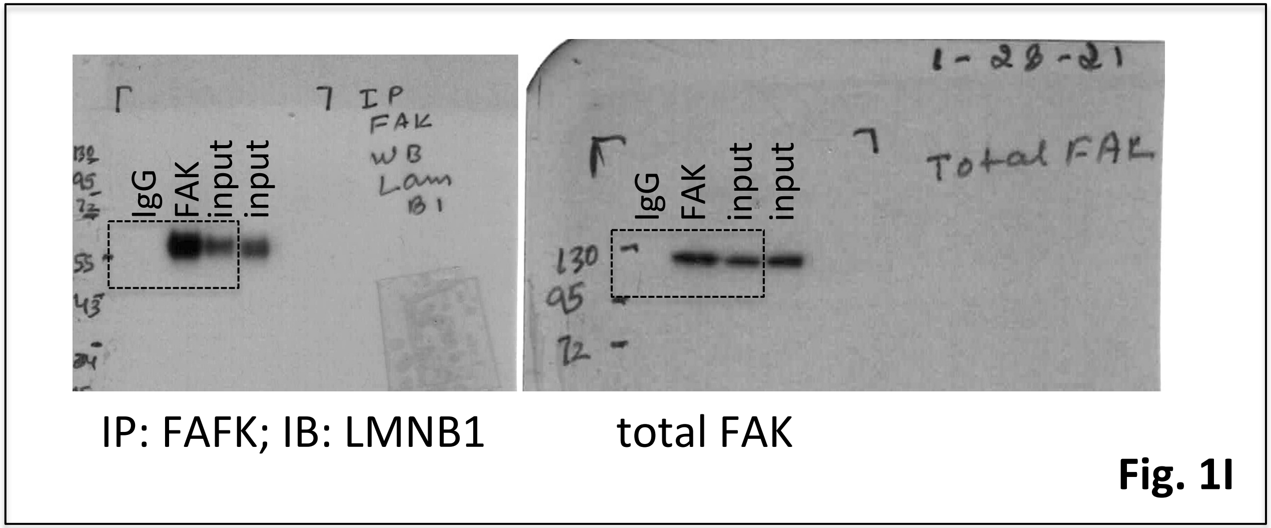
IgG
FAK
input
input
IgG
FAK
input
input
130
95
72
IP: FAFK; IB: LMNB1
total FAK

Fig. 1I

Fig. 2A

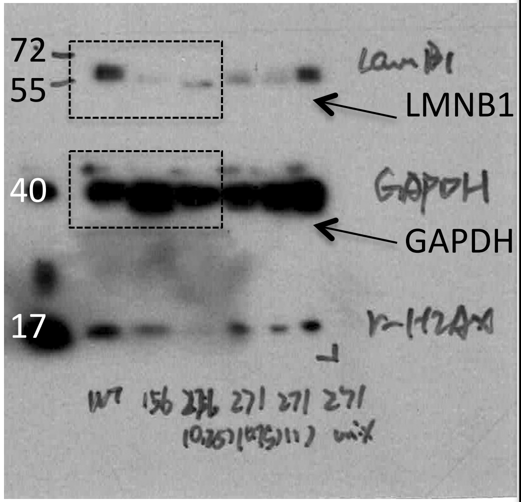

shLMNB1
(156, 272, 271, 271)

Fig. 2D

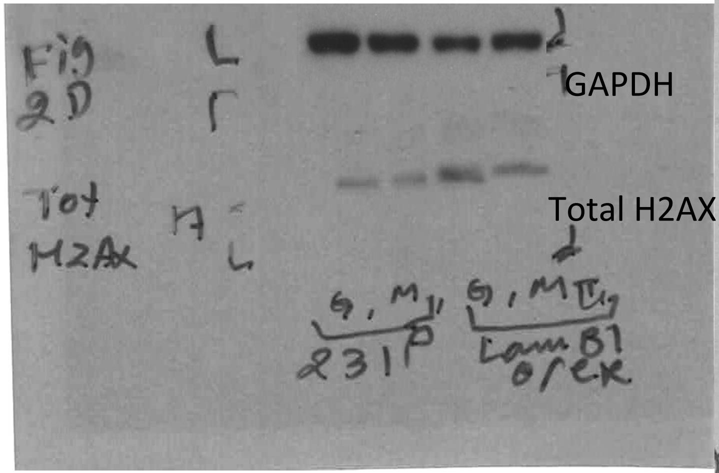

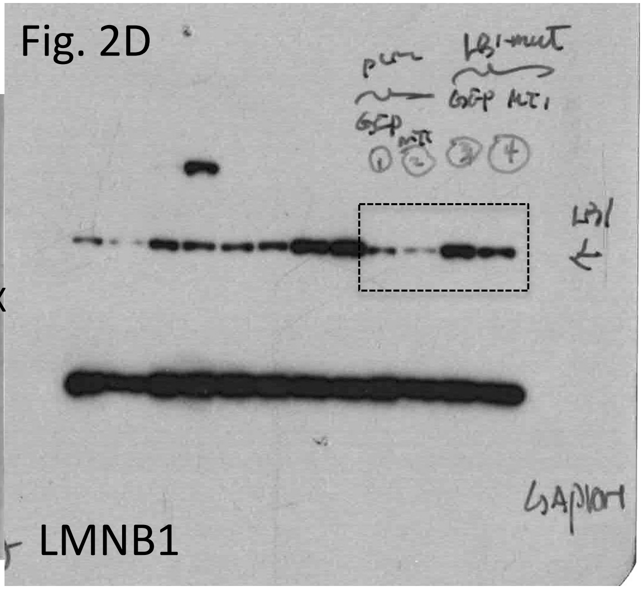

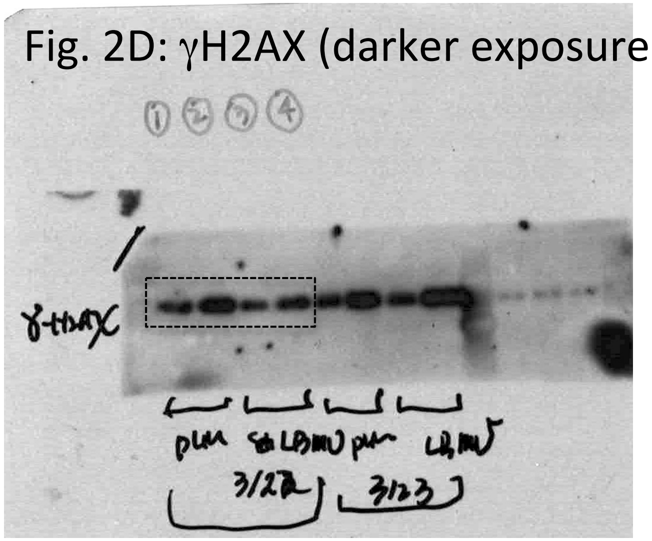

1: shGFP, pLM; 2: shMT1, pLM
3: shGFP, LMNB1; 4: shMT1, LMNB1

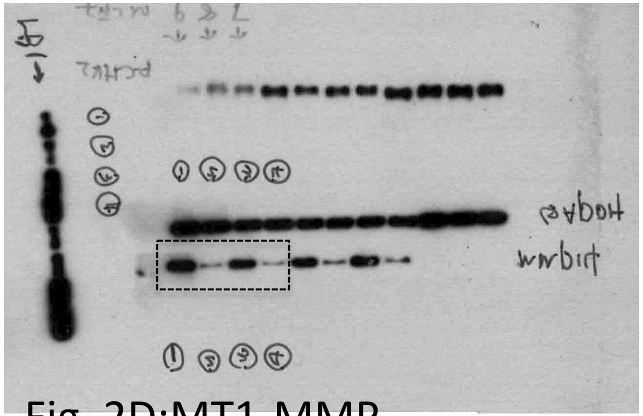

Fig. 2D:MT1-MMP

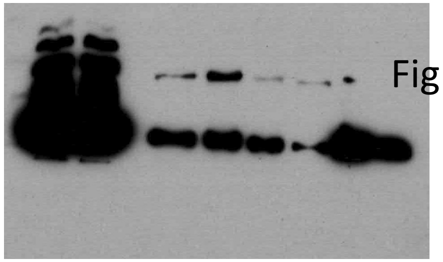

Fig. 2D:pRPA32

Fig 4 upper panel

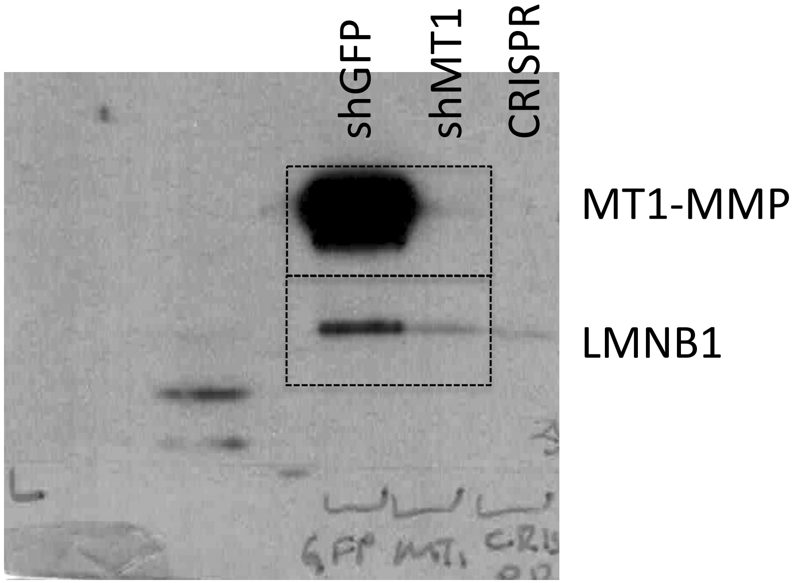

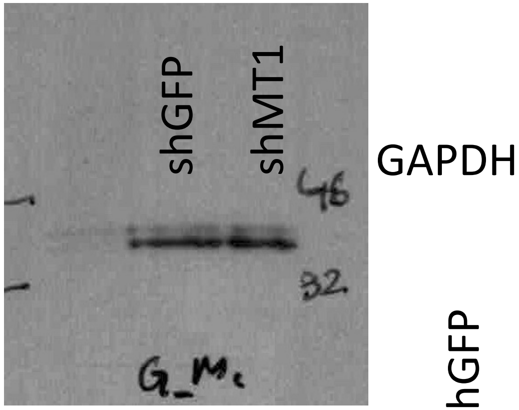

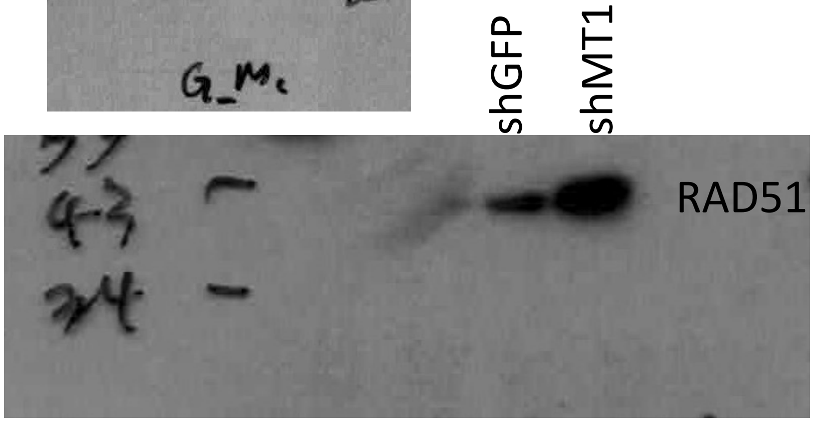

Fig. 4 bottom panel

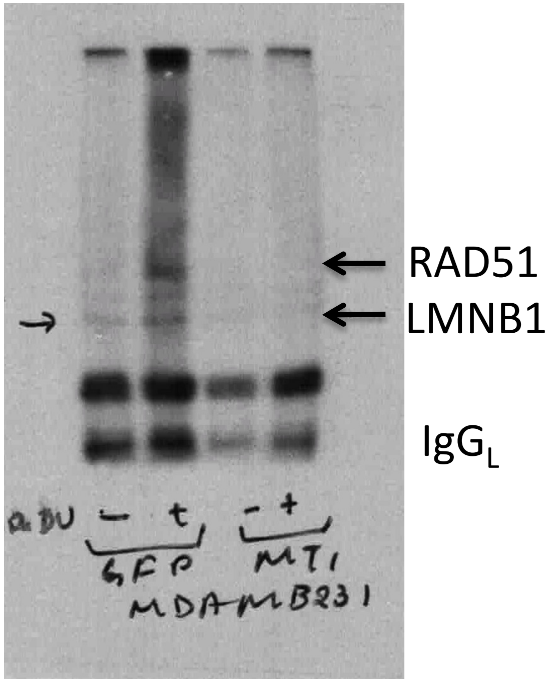

Supplement: S1 Raw images — (PDF) [file pone.0253062.s005.pdf]
